# Efficacy and Safety of Parathyroid Hormone Replacement With TransCon PTH in Hypoparathyroidism: 26‐Week Results From the Phase 3 PaTHway Trial

**DOI:** 10.1002/jbmr.4726

**Published:** 2022-11-12

**Authors:** Aliya A Khan, Mishaela R Rubin, Peter Schwarz, Tamara Vokes, Dolores M Shoback, Claudia Gagnon, Andrea Palermo, Claudio Marcocci, Bart L Clarke, Lisa G Abbott, Lorenz C Hofbauer, Lynn Kohlmeier, Susanne Pihl, Xuebei An, Walter Frank Eng, Alden R Smith, Jenny Ukena, Christopher T Sibley, Aimee D Shu, Lars Rejnmark

**Affiliations:** ^1^ Endocrinology, Metabolism, and Geriatrics McMaster University Hamilton Ontario Canada; ^2^ Endocrinology Columbia University New York New York USA; ^3^ Internal Medicine and Endocrinology Rigshospitalet Copenhagen Denmark; ^4^ Endocrinology, Diabetes, and Metabolism University of Chicago Chicago Illinois USA; ^5^ Endocrinology UCSF/VA Medical Center San Francisco California USA; ^6^ CHU de Québec‐Université Laval Research Centre and Department of Medicine Université Laval Quebec City Quebec Canada; ^7^ Unit of Metabolic Bone and Thyroid Disorders, Fondazione Policlinico Campus Bio‐medico, and Unit of Endocrinology and Diabetes Campus Bio‐medico University Rome Italy; ^8^ Endocrinology University di Pisa Pisa Italy; ^9^ Endocrinology Mayo Clinic E18‐A Rochester Minnesota USA; ^10^ Northern Nevada Endocrinology University of Nevada Reno Nevada USA; ^11^ Endocrinology, Diabetes, and Metabolic Bone Diseases Technische Universität Dresden Medical Center Dresden Germany; ^12^ Endocrinology Endocrinology and Spokane Osteoporosis Spokane Washington USA; ^13^ Biolanalysis and Pharmacokinetics/Pharmacodynamics Ascendis Pharma A/S Hellerup Denmark; ^14^ Endocrine Medical Sciences Ascendis Pharma Inc Palo Alto California USA; ^15^ Clinical Medicine and Endocrinology Aarhus University Hospital Aarhus Denmark

**Keywords:** CLINICAL TRIALS, DISORDERS OF CALCIUM/PHOSPHATE, HORMONE REPLACEMENT, PARATHYROID‐RELATED DISORDERS, PTH/VIT D/FGF23

## Abstract

Conventional therapy for hypoparathyroidism consisting of active vitamin D and calcium aims to alleviate hypocalcemia but fails to restore normal parathyroid hormone (PTH) physiology. PTH replacement therapy is the ideal physiologic treatment for hypoparathyroidism. The double‐blind, placebo‐controlled, 26‐week, phase 3 PaTHway trial assessed the efficacy and safety of PTH replacement therapy for hypoparathyroidism individuals with the investigational drug TransCon PTH (palopegteriparatide). Participants (*n* = 84) were randomized 3:1 to once‐daily TransCon PTH (initially 18 μg/d) or placebo, both co‐administered with conventional therapy. The study drug and conventional therapy were titrated according to a dosing algorithm guided by serum calcium. The composite primary efficacy endpoint was the proportion of participants at week 26 who achieved normal albumin‐adjusted serum calcium levels (8.3–10.6 mg/dL), independence from conventional therapy (requiring no active vitamin D and ≤600 mg/d of calcium), and no increase in study drug over 4 weeks before week 26. Other outcomes of interest included health‐related quality of life measured by the 36‐Item Short Form Survey (SF‐36), hypoparathyroidism‐related symptoms, functioning, and well‐being measured by the Hypoparathyroidism Patient Experience Scale (HPES), and urinary calcium excretion. At week 26, 79% (48/61) of participants treated with TransCon PTH versus 5% (1/21) wiplacebo met the composite primary efficacy endpoint (*p* < 0.0001). TransCon PTH treatment demonstrated a significant improvement in all key secondary endpoint HPES domain scores (all *p* < 0.01) and the SF‐36 Physical Functioning subscale score (*p* = 0.0347) compared with placebo. Additionally, 93% (57/61) of participants treated with TransCon PTH achieved independence from conventional therapy. TransCon PTH treatment normalized mean 24‐hour urine calcium. Overall, 82% (50/61) treated with TransCon PTH and 100% (21/21) wiplacebo experienced adverse events; most were mild (46%) or moderate (46%). No study drug‐related withdrawals occurred. In conclusion, TransCon PTH maintained normocalcemia while permitting independence from conventional therapy and was well‐tolerated in individuals with hypoparathyroidism. © 2022 The Authors. *Journal of Bone and Mineral Research* published by Wiley Periodicals LLC on behalf of American Society for Bone and Mineral Research (ASBMR).

## Graphical abstract

TransCon PTH is an investigational once‐daily prodrug providing sustained release of active PTH designed to maintain stable PTH levels in the physiological range for 24 hours/day. The PaTHway trial met all primary and key secondary endpoints with statistically significant differences from placebo. Most adverse events (AEs) were mild/moderate. Injection‐site reactions and headaches were the most common treatment‐related AEs.

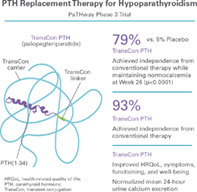

## Introduction

Hypoparathyroidism is an endocrine disease caused by insufficient or absent production of parathyroid hormone (PTH) with multiorgan involvement. Under normal physiological conditions, PTH and its downstream hormone calcitriol are the primary regulators of calcium and phosphate and act on the bone, kidney, and intestine.^(^ ^1^, ^2^ ^)^ Hypoparathyroidism can lead to hypocalcemia and hyperphosphatemia.^(^ ^1^ ^)^ As a result, individuals with hypoparathyroidism may experience a range of severe and potentially life‐threatening short‐term and long‐term complications, including neuromuscular irritability, renal complications, and vascular calcifications.^(^ ^1^, ^2^, ^3^ ^)^ Cognitive impairment, perhaps independent of abnormalities in calcium levels, is also common.^(^ ^4^, ^5^, ^6^ ^)^ All of these are associated with reduced health‐related quality of life (HRQoL).^(^ ^7^ ^)^ The negative impact of these hypoparathyroidism symptoms on daily life and work productivity is substantial^(^ ^8^, ^9^ ^)^ and underrecognized, despite published studies highlighting them.

Current consensus guidelines for the long‐term management of chronic hypoparathyroidism have identified six therapeutic goals: (i) prevent signs and symptoms of hypocalcemia; (ii) maintain the serum calcium concentration within or slightly below the normal range; (iii) maintain the calcium × phosphate product below 55 mg^2^/dL^2^ (4.4 mmol^2^/L^2^); (iv) avoid hypercalciuria; (v) avoid hypercalcemia; and (vi) avoid renal (nephrolithiasis/nephrocalcinosis) and other extraskeletal calcifications.^(^ ^3^ ^)^ Conventional therapy for hypoparathyroidism consists of active vitamin D (e.g., calcitriol or its analog alfacalcidol) and large quantities of calcium, and is associated with a substantial pill burden.^(^ ^10^ ^)^ Although conventional therapy targets hypocalcemia and its acute symptoms, it fails to restore the normal physiological effects of PTH. Additionally, long‐term conventional therapy may increase the filtered load of calcium and further elevate serum phosphorus thereby increasing the risk of nephrolithiasis, nephrocalcinosis, and chronic kidney disease.^(^ ^1^, ^11^ ^)^ Conventional therapy also does not improve HRQoL^(^ ^7^ ^)^ or the functional and mental health status of individuals with hypoparathyroidism.^(^ ^8^, ^12^, ^13^ ^)^

PTH replacement therapy is the ideal physiologic treatment for hypoparathyroidism.^(^ ^14^, ^15^ ^)^ Adjunct PTH therapy via subcutaneous (SC) injection of recombinant human PTH(1‐84) has demonstrated efficacy in achieving ≥50% reduction of active vitamin D and oral calcium while maintaining albumin‐corrected serum calcium at or above baseline levels and within the range of 7.5–10.6 mg/dL (1.87–2.64 mmol/L).^(^ ^16^, ^17^ ^)^ Evidence from a small, randomized crossover trial in adults with postsurgical hypoparathyroidism showed that continuous infusion (via insulin pump) of PTH(1‐34), in contrast to twice‐daily administration, decreased both urine calcium excretion and the cumulative dose required to maintain normocalcemia, all in the absence of conventional therapy.^(^ ^18^ ^)^ There remains an unmet need for PTH replacement therapy capable of restoring physiological levels and functions of PTH, improving HRQoL, and reducing the symptom burden and long‐term complications of hypoparathyroidism.

TransCon PTH (palopegteriparatide) is an investigational once‐daily prodrug with sustained release of active PTH in development as a treatment for adults with hypoparathyroidism.^(^ ^19^, ^20^ ^)^ TransCon PTH consists of a parent drug, PTH(1‐34), transiently conjugated to a branched methoxypolyethylene glycol (mPEG) carrier through a proprietary linker. It is administered as a once‐daily SC injection designed to provide stable PTH levels within the normal physiological range over 24 h/d.^(^ ^13^, ^19^, ^20^ ^)^ In the phase 2 PaTH Forward trial, daily TransCon PTH enabled independence from oral active vitamin D and therapeutic doses of elemental calcium (>500 mg/d) in 91% of participants. Participants treated with TransCon PTH also achieved normal mean serum calcium, serum phosphate, and urine calcium, and demonstrated improved HRQoL over 26 weeks of treatment.^(^ ^13^ ^)^ The primary objective of the phase 3, randomized, double‐blind, placebo‐controlled PaTHway trial was to assess the treatment effect of once‐daily TransCon PTH on serum calcium levels and therapeutic doses of active vitamin D (ie, calcitriol or alfacalcidol) and elemental calcium in adults with hypoparathyroidism over 26 weeks. Additional objectives were to assess the effect of TransCon PTH on HRQoL, symptom improvement, functioning, well‐being, and urinary calcium excretion in this population.

## Materials and Methods

### Trial design

PaTHway is a phase 3, multicenter (North America and Europe), randomized, double‐blind, placebo‐controlled, parallel‐group, 26‐week trial with an open‐label extension of 156 weeks that evaluated the efficacy, safety, and tolerability of once‐daily TransCon PTH as PTH replacement therapy in individuals with hypoparathyroidism. Screening for the trial began on February 15, 2021. The last participant completed the blinded portion of the trial on January 12, 2022, and the open‐label extension is ongoing. The trial consisted of an approximately 4‐week screening period followed by a 26‐week blinded treatment period. All participants who completed the 26‐week blinded treatment period were allowed to enroll in the 156‐week open‐label extension period. Data through week 26 of the PaTHway trial are reported herein. The protocol was reviewed by the appropriate institutional review boards and independent ethics committees (ClinicalTrials.gov identifier: NCT04701203; EudraCT no: 2020‐003380‐26). The trial was designed by the sponsor and authors and conducted in accordance with the principles of the Declaration of Helsinki and Good Clinical Practice guidelines as described in the International Conference on Harmonization Guideline E6.

### Participants

Eligible participants included men and nonpregnant women (≥18 years of age) with chronic hypoparathyroidism of postsurgical, autoimmune, genetic, or idiopathic etiologies for a duration of at least 26 weeks. Participants must have been treated with calcitriol ≥0.5 μg/d or alfacalcidol ≥1.0 μg/d in addition to elemental calcium ≥800 mg/d for at least 12 weeks before screening. Stable doses of conventional therapy (ie, active vitamin D and calcium) were required for at least 5 weeks before screening, not precluding occasional (≤2/week) pro re nata (PRN, as needed) doses of calcium and/or active vitamin D. To ensure safety for those who might be randomized to the placebo arm, participants were required to achieve an albumin‐adjusted or ionized serum calcium within the normal range or slightly below the normal range (albumin‐adjusted serum calcium 7.8–10.6 mg/dL [1.95–2.64 mmol/L] or ionized serum calcium 4.40–5.29 mg/dL [1.10–1.32 mmol/L]), 25(OH) vitamin D levels 20–80 ng/mL (49–200 nmol/L), and magnesium levels ≥1.3 mg/dL (0.53 mmol/L), as is currently the goal of conventional treatment.^(^ ^3^ ^)^ Urinary calcium excretion ≥125 mg/24 h and estimated glomerular filtration rate (eGFR) ≥30 mL/min/1.73 m^2^ was required for enrollment. The diagnosis of hypoparathyroidism was confirmed by the presence of persistent hypocalcemia in the setting of inappropriately low serum PTH levels. Low PTH levels were defined as at or below the median value of the reference range at the performing laboratory. Individuals with impaired responsiveness to PTH (pseudohypoparathyroidism) or any disease that might affect calcium metabolism, calcium × phosphate homeostasis, or PTH levels other than hypoparathyroidism were excluded. Thiazide diuretic use was not allowed within 4 weeks before the 24‐hour urine collection before visit 1, and the use of PTH analogs was not allowed within 4 weeks of screening. Eligible participants could not have used other drugs known to influence calcium and bone metabolism (except for active vitamin D analogs and elemental calcium) within 12 weeks before screening or osteoporosis therapies known to influence calcium and bone metabolism (ie, bisphosphonate) within 2 years before screening. The use of loop diuretics, phosphate binders, digoxin, lithium, methotrexate, biotin >30 μg/d, and systemic corticosteroids (other than as replacement therapy) were not permitted during the trial. All participants provided signed informed consent before treatment initiation.

### Trial protocol

Once enrolled, participants were randomized 3:1 into two treatment groups: TransCon PTH 18 μg PTH(1‐34)/d or placebo (excipient solution) mimicking 18 μg/d, both co‐administered with conventional therapy (active vitamin D and elemental calcium). Randomization was stratified by etiology of hypoparathyroidism (postsurgical versus nonsurgical). In addition to frequent laboratory visits to measure serum calcium levels, the blinded treatment period included 10 clinic visits over 26 weeks. Once‐daily treatment with TransCon PTH or placebo was delivered by a modified Ypsomed (Burgdorf, Switzerland) UnoPen Fix pen injector using 31‐gauge, 5‐mm pen needles to deliver doses of 6–30 μg/injection in a volume of ≤100 μL to either abdomen or anterior thigh, rotating injection sites. All participants were initially prescribed TransCon PTH 18 μg PTH(1‐34)/d or a corresponding volume of placebo and were individually and progressively titrated to an optimal dose (allowable range 6–60 μg/d) in increments of 3 μg/d. Titration of study drug and conventional therapy was performed according to a protocol‐specified algorithm guided by serum calcium values (Supplemental Fig. SS1). The algorithm was intended to facilitate independence from conventional therapy by discontinuation of active vitamin D and calcium in response to reestablishment of physiological PTH signaling with TransCon PTH treatment. In the setting of normocalcemia, the protocol directs incremental increases in study drug (TransCon PTH or placebo) accompanied by stepwise reductions of active vitamin D by 33% to 50% of the starting dose, followed by reductions of calcium in at least 1500‐mg/d increments once vitamin D has been discontinued. All doses >30 μg/d were administered in the form of two sequential doses injected at different injection sites using two pens. Injections were self‐administered after training by trial staff. Participants were permitted to take elemental calcium ≤600 mg/d as a nutritional supplement if required to meet the generally recommended dietary intakes of calcium.

### Efficacy assessments

The composite primary efficacy outcome of the PaTHway trial was the proportion of participants at week 26 who achieved albumin‐adjusted serum calcium in the normal range (8.3–10.6 mg/dL [2.07–2.64 mmol/L]), independence from active vitamin D, and independence from therapeutic doses of elemental calcium (>600 mg/d) with no increase in the prescribed study drug over the 4 weeks before week 26. Independence from active vitamin D was defined as a daily standing dose equal to zero on all days and use of any PRN active vitamin D on no more than 7 days during the 4 weeks before the week 26 visit. Independence from therapeutic calcium was defined as an average daily standing dose of ≤600 mg and use of PRN doses on no more than 7 days during the 4 weeks before the week 26 visit. Secondary efficacy outcomes included active vitamin D and elemental calcium doses, daily “pill burden” of active vitamin D and calcium (as oral tablets, powder, liquid solutions, liquid suspensions, or transdermal patches), albumin‐adjusted serum calcium, serum phosphate, and serum calcium × phosphate product.

### Patient‐reported outcomes

At baseline and weeks 10, 20, and 26, participants completed the Hypoparathyroidism Patient Experience Scale (HPES), a psychometrically validated, disease‐specific questionnaire that includes both symptom and impact measures.^(^ ^21^ ^)^ The 17‐item HPES‐Symptom assesses key hypoparathyroidism‐related physical and cognitive symptoms from the patient perspective, and the 26‐item HPES‐Impact assesses the impact of these symptoms also from the patient perspective on patient functioning and well‐being, such as physical functioning, daily life, psychological well‐being, social life, and relationships. Participants were asked to rate the frequency of each symptom as never (0%), occasionally (1%–25%), sometimes (26%–50%), often (51%–75%), or very often/always (76%–100%), with answers scored 0 to 4, respectively. For questions within a particular subcategory or domain, answers were averaged to create a domain‐level score. Domain‐level scores were then averaged to create a total score. Finally, domain level and total scores were both transformed into a 0 to 100 scale, with lower scores indicating improvement.

Participants also completed the 36‐Item Short‐Form Health Survey (SF‐36, version 2), a 36‐item health questionnaire that assesses eight dimensions of health: physical functioning, role physical (ie, role limitations due to physical health problems), bodily pain, general health, vitality, social functioning, role emotional (ie, role limitations due to emotional problems), and mental health. The SF‐36 also includes two summary scores for physical and mental health. All scores are expressed relative to the mean of the general US population, with higher scores corresponding to less disability.^(^ ^22^ ^)^ Scores for each of the eight subscales were averaged to create norm‐based domain scores. The change from baseline to week 26 in HPES‐Symptom physical domain score, HPES‐Symptom cognitive domain score, HPES‐Impact physical functioning domain score, HPES‐Impact daily life domain score, and SF‐36 physical functioning subscale were key secondary endpoints.

### Safety assessments

At prespecified intervals throughout the trial, information on the use of concomitant medications was collected. Serum chemistries, hematology, and 25‐hydroxyvitamin D levels, as well as antibodies against PTH, TransCon PTH, and polyethylene glycol (PEG) were measured. Twenty‐four‐hour urine calcium excretion was also assessed. Clinical events of symptomatic hypo‐ or hypercalcemia were also monitored. Treatment‐emergent adverse events (TEAEs), adverse events of special interest (AESI), and treatment‐emergent serious adverse events (SAEs) were documented by site staff at clinic visits and upon review of participant diaries. The seriousness, severity, and causality of all recorded TEAEs were assessed by the trial investigators. Vasodilatory signs and symptoms were prespecified as AESIs.

### Statistical analysis

Sample size calculations assuming a 70% response rate for TransCon PTH and 15% for placebo for the primary composite endpoint indicated that 68 participants randomized 3:1 to active TransCon PTH versus placebo would have 99% statistical power at *α* equal to 0.05 to demonstrate a statistically significant difference between the two treatments. Accounting for a ~10% dropout rate, a total sample size of 76 participants was targeted. All efficacy analyses were done using the intent‐to‐treat (ITT) population, which consisted of all participants who were randomized and received at least one dose of the blinded study drug. Efficacy analyses were based on treatment assignment per randomization. At each post‐baseline visit, only data from participants with both baseline and the corresponding visit values available were used to compute the statistical summaries. The Safety Analysis Population consisted of all randomized participants who received at least one dose of the study drug; safety analyses were based on the Safety Analysis Population and actual treatment received.

Data from clinical assessments were summarized using descriptive statistics. Categorical data were presented using counts and percentages of participants, whereas continuous variables were summarized by mean and standard deviation (SD). All statistical tests were 2‐sided and tested at the statistically significant level of *α* equal to 0.05. Confidence intervals were 2‐sided 95% confidence intervals unless stated otherwise.

For the primary efficacy endpoint, the Cochran–Mantel Haenszel test stratified by etiology of hypoparathyroidism (postsurgical or other) was used to compare the proportion of participants meeting the composite primary endpoint in the TransCon PTH versus placebo groups. A prespecified sensitivity analysis of the primary composite endpoint permitting no PRN doses of active vitamin D or calcium for 4 weeks before the week 26 visit was also performed. Participants without week 26 albumin‐adjusted serum calcium or with >25% (ie, >7 days) missing diary data of active vitamin D or elemental calcium during the 4 weeks before week 26 were considered non‐responders.

Continuous secondary endpoints were analyzed using an analysis of covariance (ANCOVA) model with unequal variance, which included change from baseline for the endpoint of interest as a response variable, treatment assignment and etiology of hypoparathyroidism as fixed factors, and baseline variable of the endpoint as a covariate. For key secondary patient‐reported outcomes (PROs), the ANCOVA model described above was used to test the change from baseline to week 26 between participants treated with TransCon PTH versus placebo in a prespecified sequence. The prespecified sequential testing procedure was applied to control the family‐wise, type 1 error rate for the primary and key secondary endpoints. All statistical analyses were conducted using SAS version 9.4 (SAS Institute, Cary, NC, USA).

## Results

### Participant disposition and baseline demographics

**Fig. 1:**
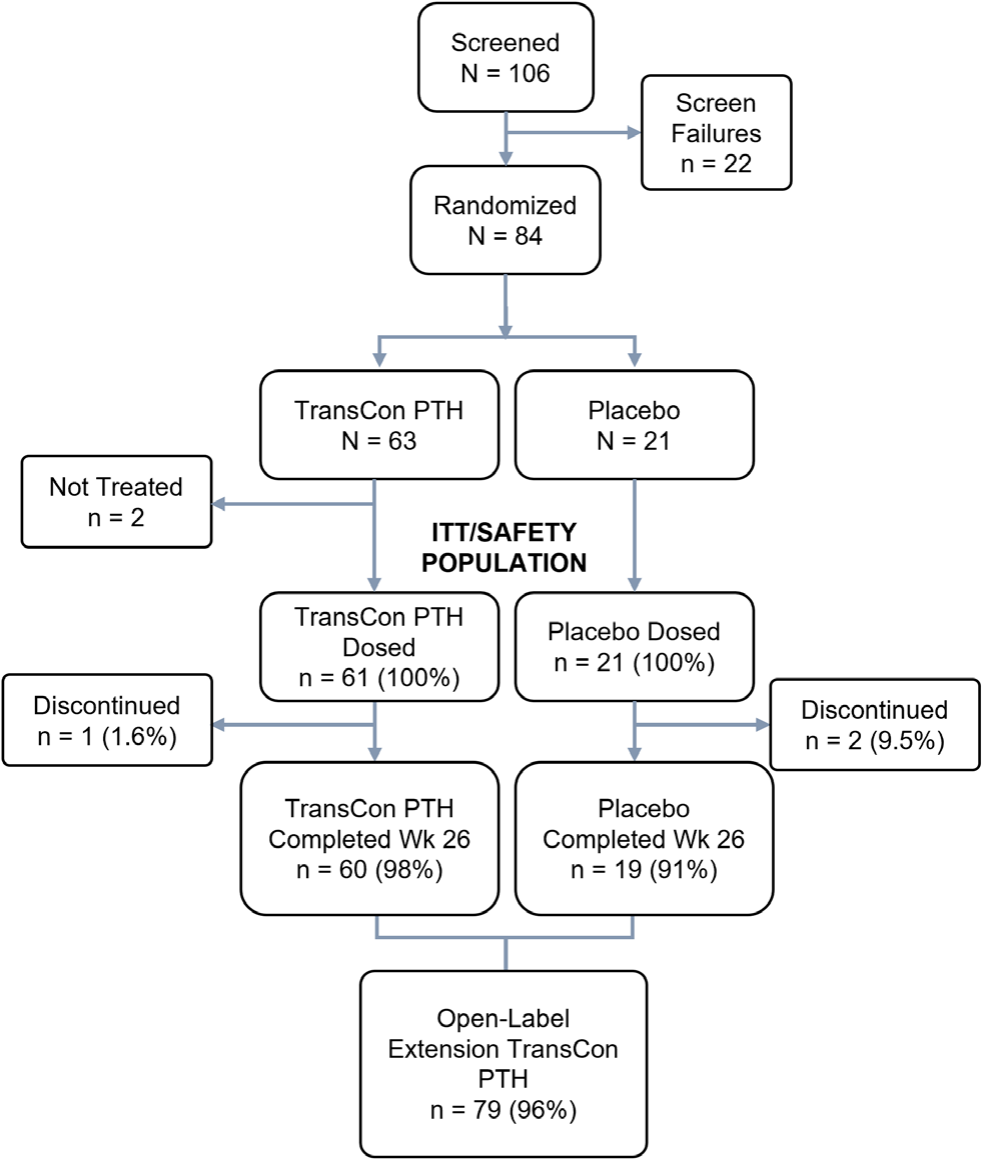
Patient disposition. A total of 106 participants were screened, and 84 met eligibility criteria, were enrolled in the trial, and randomized to treatment. Two participants randomized to TransCon PTH discontinued the trial before receiving treatment: one withdrew consent, and one withdrew due to thyroid cancer recurrence. Sixty‐one participants in the TransCon PTH and 21 participants in the placebo group received ≥1 blinded treatment and comprised the intent‐to‐treat population. During the trial, one participant in the TransCon PTH group experienced a fatal cardiac arrest (unrelated to the study drug), and 2 withdrew from the placebo group: one withdrew consent, and one withdrew due to breast cancer. A total of 79 participants completed the blinded treatment through week 26.

A total of 106 participants were screened for eligibility, and 84 were randomized to treatment (*n* = 63 TransCon PTH, *n* = 21 placebo; Fig. 1). Of those randomized, 82 received at least one dose of the study drug (*n* = 61 TransCon PTH, *n* = 21 placebo), and 79 completed the blinded treatment through week 26 (*n* = 60 TransCon PTH, *n* = 19 placebo). The treatment groups were balanced with respect to age, sex, race, and baseline hypoparathyroidism characteristics (Table 1). The mean age of the study population was 48.6 years, 78% were female (*n* = 64), and 93% were White (*n* = 76). Mean (SD) duration of hypoparathyroidism was 11.7 (10.7) years. Seventy participants (85.4%) had postsurgical hypoparathyroidism. Of the 12 participants with other etiologies, 7 were diagnosed with primary idiopathic disease, 2 autoimmune polyglandular syndrome type 1 (APS‐1), 1 autosomal dominant hypocalcemia (activating mutation of the calcium‐sensing receptor [CaSR]), 1 HDR syndrome (hypoparathyroidism, sensorineural deafness, and renal disease; also known as Barakat syndrome), and 1 with DiGeorge syndrome. At baseline, the mean (SD) total daily dose of calcitriol was 0.75 (0.34) μg (*n* = 70), alfacalcidol 2.33 (0.78) μg (*n* = 12), and elemental calcium 1839.4 (1049.6) mg (*n* = 82).

**Table 1:** Baseline Demographics, Disease Characteristics, and Supplementation

Characteristics	TransCon PTH (*n* = 61)	Placebo (*n* = 21)
Age (years), mean (SD)	49.0 (13.1)	47.3 (11.4)
Age group (years), *n* (%)		
<50	28 (46)	14 (67)
≥50	33 (54)	7 (33)
Sex at birth, female *n* (%)	46 (75)	18 (86)
Postmenopausal, *n* (%)	19 (41)	3 (17)
Body mass index (kg/m^2^), mean (SD)	27.3 (5.8)	29.5 (5.7)
Race, *n* (%)		
Asian	3 (5)	2 (10)
White	57 (93)	19 (91)
Other	1 (2)	0
Geographic region, *n* (%)		
North America	39 (64)	12 (57)
Europe	22 (36)	9 (43)
Hypoparathyroidism etiology, *n* (%)		
Acquired from neck surgery	52 (85)	18 (86)
Autoimmune disease	1 (2)	0
Intrinsic genetic defects of the parathyroid glands	3 (5)	0
Idiopathic disease	4 (7)	3 (14)
Other	1 (2)	0
Duration of hypoparathyroidism (years), mean (SD)	12.0 (11.4)	11.1 (8.5)
Baseline conventional therapy total daily doses, mean (SD)		
Elemental calcium (mg)	1748 (904)	2105 (1383)
Calcitriol (μg)	0.76 (0.34)	0.69 (0.33)
Alfacalcidol (μg)	2.5 (0.9)	2.0 (0.4)
Pill burden (active vitamin D and calcium), mean (SD)	6.7 (2.2)	6.7 (3.0)
24‐hour urine calcium (mg/day), mean (SD)	392 (175)	329 (140)

Abbreviations: PTH = parathyroid hormone; SD = standard deviation.

### Efficacy

**Fig. 2:**
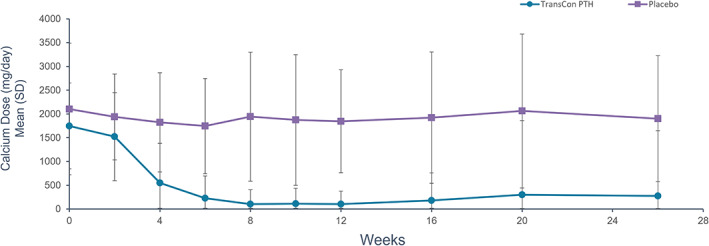
Elemental calcium supplement dose with TransCon PTH treatment. Treatment with TransCon PTH resulted in a greater reduction in mean daily calcium dose than placebo as early as 4 weeks (*p* = 0.0003) and through 26 weeks (*p* = 0.0003). After 4 weeks of treatment with TransCon PTH, elemental calcium intake decreased from a baseline mean (SD) of 1748.0 (903.9) mg/d to a mean (SD) dose of 548.8 (832.7) mg/d, which continued to decrease throughout the trial to a mean (SD) dose of 274.2 (1371.8) mg/d at week 26. Per trial protocol, participants were permitted to take calcium ≤600 mg/d as a nutritional supplement, if needed, to meet the recommended dietary intake of calcium. Negative error bars (SD) are not displayed for values less than zero.

**Fig. 3:**
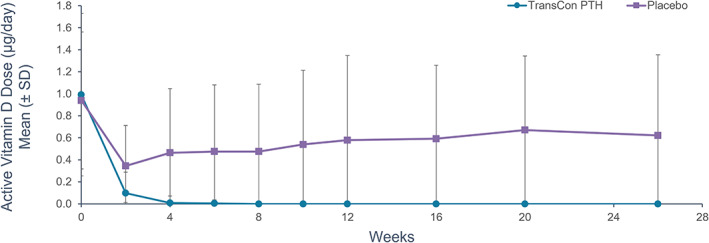
Active vitamin D supplement dose with TransCon PTH treatment. Per trial protocol, all participants decreased their active vitamin D dose by 33% to 50% (eg, by skipping the second dose of the day if taking 2 times daily (BID), skipping the final dose of the day if taking 3 times daily (TID), or reducing a once‐daily dose of alfacalcidol ≥1.0 μg by 50% (≥0.5 μg) at the start of the blinded treatment period. Subsequent dose decreases or discontinuations were done according to a predefined protocol. Within 4 weeks, the majority of participants treated with TransCon PTH discontinued active vitamin D. Mean doses of active vitamin D supplements were calculated from actual micrograms prescribed and are not adjusted for relative potency of calcitriol versus alfacalcidol. The difference in least squares (LS) means between TransCon PTH and placebo groups was statistically significant at all time points (*p* < 0.0001). Negative error bars (SD) are not displayed for values less than zero.

**Fig. 4:**
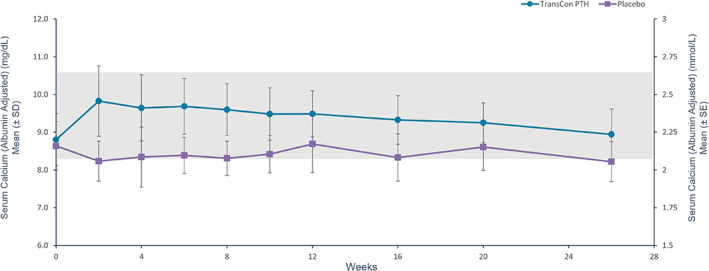
Albumin‐adjusted serum calcium with TransCon PTH treatment. In the TransCon PTH group, mean serum calcium values remained within the normal range at all study visits through week 26. Baseline mean serum calcium was 8.8 mg/dL (2.2 mmol/L) and 8.6 mg/dL (2.15 mmol/L) for TransCon PTH and placebo, respectively, and 8.9 mg/dL (2.22 mmol/L) and 8.2 mg/dL (2.05 mmol/L), respectively, at week 26. Normal range for serum calcium = 8.3–10.6 mg/dL (2.07–2.64 mmol/L).

After 26 weeks of blinded treatment, 79% (48/61) of participants receiving TransCon PTH versus 5% (1/21) receiving placebo achieved the primary composite endpoint of independence from conventional therapy, with maintenance of normocalcemia without an increase in study drug dose in the final 4 weeks of the blinded period (*p* < 0.0001) (Table 2). A prespecified sensitivity analysis permitting no PRN doses of active vitamin D or calcium in the final 4 weeks showed similar results, with 74% (45/61) of participants receiving TransCon PTH and 5% (1/21) receiving placebo meeting the composite sensitivity analysis endpoint (*p* < 0.0001). Ninety‐three percent (57/61) of participants treated with TransCon PTH achieved independence from conventional therapy. Treatment with TransCon PTH enabled rapid and sustained reduction of elemental calcium (Fig. 2) and complete discontinuation of active vitamin D within 8 weeks (Fig. 3). At week 26, the mean TransCon PTH dose in participants allocated to active therapy was 21.4 μg/d (median 21 μg/d, range 9–39 μg/d). Daily pill burden decreased from a mean (SD) of 6.7 (2.2) in the TransCon PTH group at baseline to 0.5 (1.7) at week 26, and from 6.7 (3.0) to 5.4 (3.2) in the placebo group. The least squares (LS) mean reduction in daily pill burden from baseline to week 26 was significantly greater in participants treated with TransCon PTH compared with placebo (*p* < 0.0001). Mean albumin‐adjusted serum calcium in the TransCon PTH group was normal at baseline and remained within the normal range at 26 weeks (8.8 mg/dL [2.2 mmol/L] and 8.9 mg/dL [2.22 mmol/L], respectively), whereas serum calcium declined from 8.6 mg/dL (2.15 mmol/L) at baseline to 8.2 mg/dL (2.05 mmol/L) at week 26 in the placebo group, consistent with the protocol‐mediated decrease in active vitamin D dosing in this group. Participants treated with TransCon PTH maintained mean serum calcium levels in the normal range at all study visits (Fig. 4). On average, mean serum phosphate, calcium × phosphate product, magnesium, 25‐hydroxyvitamin D, and 1,25‐dihydroxyvitamin D values were maintained within the normal range (Table 3).

**Table 2:** Percentage of Participants Meeting the Composite Primary Endpoint at Week 26

	TransCon PTH (*n* = 61)	Placebo (*n* = 21)
No. participants meeting the primary endpoint criteria at week 26 (responders)	48	1
Proportion, % (95% CI)	79% (66, 88)	5% (0.1, 24)
Hypothesis test: *p* value (TransCon PTH versus placebo)^a^	<0.0001	
No. participants meeting each component		
Albumin‐adjusted serum calcium within the normal range^b^	49	10
Independence from active vitamin D	60	5
Independence from therapeutic doses of calcium	57	1
No increase in prescribed study drug	57	12

*Note*: Participants with missing data on one or more of the criteria were considered as non‐responders.

Abbreviations: CI, confidence interval; PTH, parathyroid hormone.

^a^ Cochran–Mantel–Haenszel test controlling for etiology of hypoparathyroidism (postsurgical versus nonsurgical).

^b^ The normal range for albumin‐adjusted serum calcium is 8.3–10.6 mg/dL (2.07–2.64 mmol/L).

**Table 3:** Baseline and Week 26 Serum Biochemistries in Participants Treated With TransCon PTH and Placebo

	Baseline		Week 26	
Serum biochemistries, mean (SD)	TransCon PTH (*n* = 61)	Placebo (*n* = 21)	TransCon PTH (*n* = 60)	Placebo (*n* = 19)
Albumin‐adjusted calcium (mg/dL)	8.8 (0.7)	8.6 (0.6)	8.9 (0.7)	8.2 (0.5)
Phosphate (mg/dL)	4.2 (0.6)	3.9 (0.8)	3.8 (0.6)	3.9 (0.9)
Calcium^a^ × phosphate product (mg^2^/dL^2^)	37.1 (5.7)	33.7 (6.7)	33.9 (4.8)	31.7 (6.4)
Magnesium (mg/dL)	2.1 (0.2)	2.0 (0.2)	2.2 (0.1)	2.0 (0.2)
1,25‐Dihydroxyvitamin D (pg/mL)^b^	38.5 (11.0)	40.0 (15.4)	39.6 (19.3)	34.8 (16.5)
25‐Hydroxyvitamin D (ng/mL)^b^	43.2 (11.9)	41.1 (11.5)	36.4 (12.0)	42.2 (17.4)
eGFR (mL/min/1.73 m^2^)^c^	67.5 (13.8)	72.7 (14.6)	75.6 (14.5)	70.8 (13.4)

Abbreviation: eGFR, estimated glomerular filtration rate.

^a^ Albumin‐adjusted serum calcium.

^b^ *n* = 59 for TransCon PTH group at week 26.

^c^ eGFR adjusted for body surface area.

### Patient‐reported outcomes

**Fig. 5:**
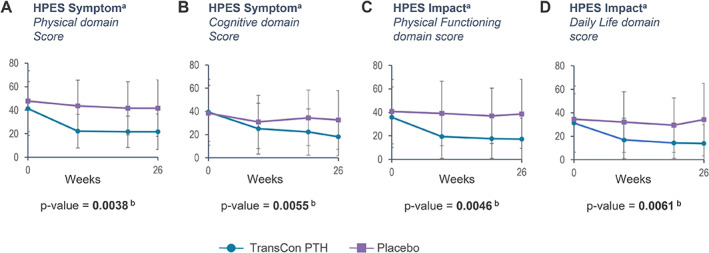
Treatment effect of TransCon PTH on the hypoparathyroidism patient experience scale (HPES).^a^The HPES is a psychometrically validated, disease‐specific measure specifically designed to assess the symptoms and impact associated with hypoparathyroidism. A higher HPES score indicates greater symptom frequency or impact. ^b^The *p* values are from ANCOVA models testing the difference in change from baseline at week 26 between TransCon PTH and placebo, with etiology of hypoparathyroidism as fixed effects and baseline HPES domain scores as covariates. TransCon PTH treatment demonstrated a significant improvement compared with placebo at week 26 in HPES‐Symptom domain scores: (*A*) physical (*p* = 0.0038) and (*B*) cognitive (*p* = 0.0055); and HPES‐Impact domain scores: (*C*) physical functioning (*p* = 0.0046) and (*D*) daily life (*p* = 0.0061). Lower HPES scores indicate improvement. Negative error bars (SD) are not displayed for values less than zero.

**Fig. 6:**
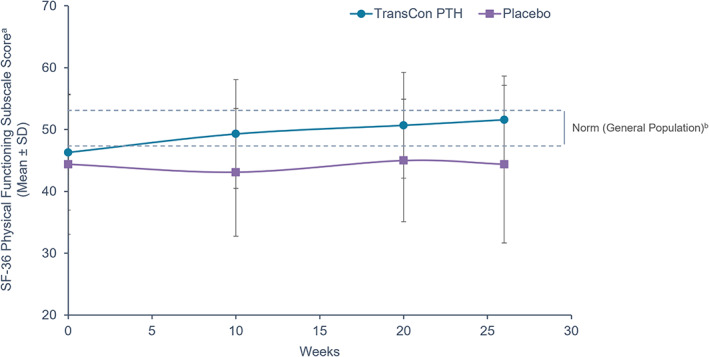
Treatment effect of TransCon PTH on general health, 36‐Item Short Form Survey (SF‐36). ^a^36‐Item Short Form Survey (SF‐36) subscale score, a patient‐reported survey that serves as a general measure of well‐being. In the SF‐36 subscale, lower scores are associated with a greater disease burden; increases in scores indicate improvement. ^b^The dashed lines indicate the upper (53) and lower (47) *T*‐score bounds for the US general population's average level of functioning, with scores below 47 indicating impairment. Source: Maruish ME (editor). User's Manual for the SF‐36v2 Health Survey (3rd ed.). Lincoln, RI: Quality Metric Incorporated.

Both HPES and SF‐36 scores improved through week 26 in the TransCon PTH group. The disease‐specific HPES showed improvements in hypoparathyroidism‐related symptoms, functioning, and well‐being for participants treated with TransCon PTH. Treatment with TransCon PTH demonstrated a statistically significant improvement compared with placebo in all key secondary endpoints at week 26: HPES‐Symptom physical (*p* = 0.0038) and cognitive (*p* = 0.0055) domain scores and HPES‐Impact physical functioning (*p* = 0.0046) and daily life (*p* = 0.0061) domain scores (Table 4; Fig. 5). Health‐related QOL as measured by the SF‐36 also improved significantly in the TransCon PTH group compared with the placebo group for the physical functioning subscale score (*p* = 0.0347 for TransCon PTH versus placebo change from baseline at week 26) (Fig. 6).

**Table 4:** Week 26 Improvement From Baseline in Hypoparathyroid Patient Experience Scale (HPES) Impact and Symptom Scores^a^

HPES scale	Total TransCon PTH (*n* = 61)	Placebo (*n* = 21)
HPES‐impact scale		
Daily life domain, *n*	59	19
Mean (SD)	−16.4 (19.6)	−2.9 (21.8)
Physical functioning domain (*n*)	59	19
Mean (SD)	−17.7 (20.4)	−5.3 (21.5)
Psychological well‐being domain (*n*)	54	17
Mean (SD)	−14.2 (19.4)	−0.9 (21.0)
Social life and relationship domain (*n*)	59	19
Mean (SD)	−13.5 (20.8)	−3.4 (21.6)
Total HPES‐impact score (*n*)	59	19
Mean (SD)	−15.7 (18.0)	−3.9 (19.0)
HPES‐symptom scale		
Physical domain (*n*)	59	19
Mean (SD)	−19.3 (17.9)	−8.4 (23.7)
Cognitive domain (*n*)	59	19
Mean (SD)	−21.0 (24.7)	−7.4 (14.2)
Total HPES‐symptom score (*n*)	59	19
Mean (SD)	−20.2 (19.5)	−7.9 (18.0)

Abbreviations: HPES, hypoparathyroid patient experience scale; PTH, parathyroid hormone; SD, standard deviation.

^a^ Lower scores reflect improvement in hypoparathyroidism‐related symptoms, functioning, and well‐being.

### Safety

**Fig. 7:**
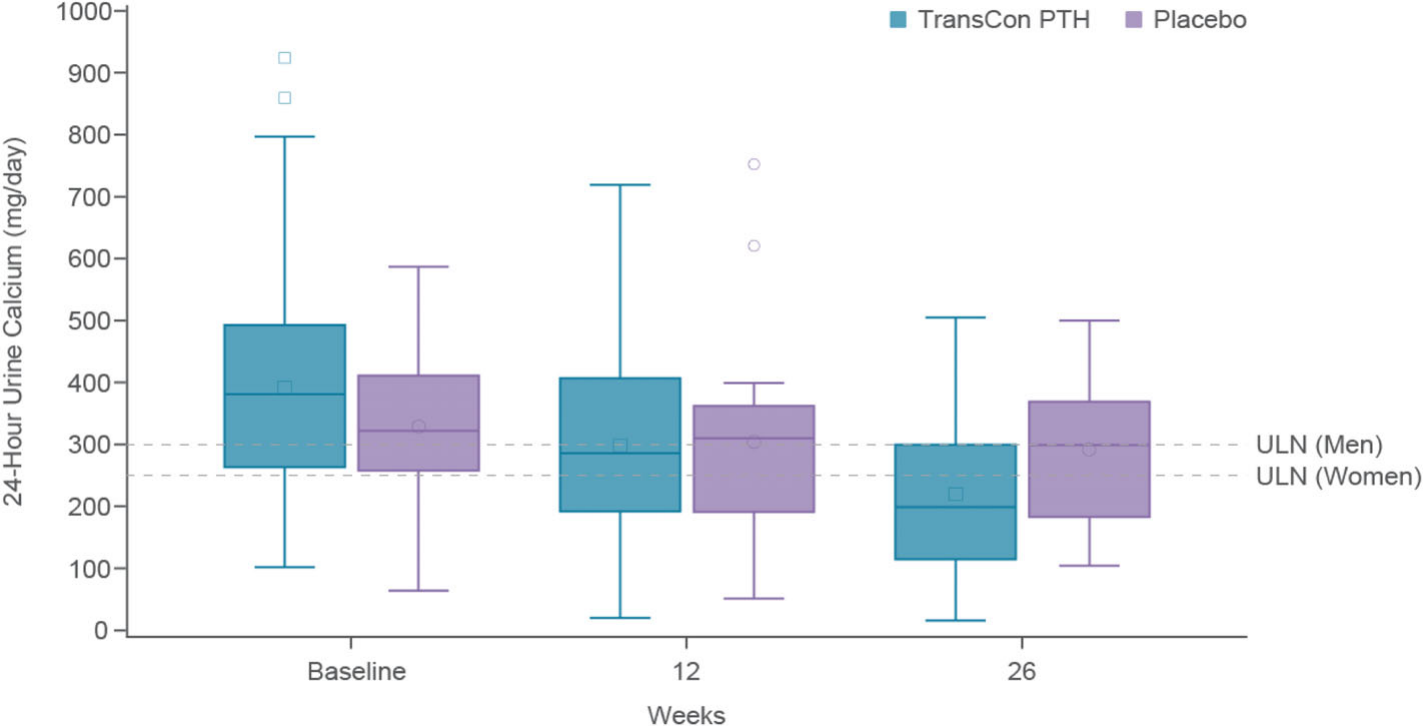
Twenty four‐hour urine calcium with TransCon PTH treatment. Box plot of 24‐hour urine calcium. Within each box, horizontal lines denote median values and the square (TransCon PTH), and circle (placebo) denote mean values; boxes extend from the 25th to the 75th percentile of each treatment group's distribution of values; vertical extending lines denote adjacent values within 1.5 interquartile range of the 25th and 75th percentile of each group; dots outside the box denote data points outside the range of adjacent values. Mean 24‐hour urine calcium values decreased from 390 mg/24 h at baseline to 220 mg/24 h at week 26 in participants treated with TransCon PTH, and from 329 to 292 mg/24 h in participants who received placebo. The mean change from baseline was statistically significant in the TransCon PTH group (*p* < 0.0001) but not in the placebo group (*p* = 0.24). An ANCOVA model testing the difference in change from baseline at week 26 between TransCon PTH and placebo, with etiology of hypoparathyroidism as fixed effects and baseline urine calcium excretion value as a covariate showed a significant difference between groups (*p* = 0.0085). ULN = upper limit of normal.

Mean 24‐hour urine calcium levels decreased from 392 mg/24 h at baseline to 220 mg/24 h in participants treated with TransCon PTH compared with 329 mg/24 h at baseline to 292 mg/24 h with placebo (Fig. 7). A greater proportion of participants treated with TransCon PTH (37/61, 60.7%) than placebo (6/21, 28.6%) achieved normal 24‐hour urine calcium excretion (≤250 mg/24 h, *p* = 0.0213). Participants treated with TransCon PTH experienced lower rates of hypocalcemia during the trial compared with placebo (Table 5).

**Table 5:** Albumin‐Adjusted Serum Calcium Excursions by Time Period

	Normal		Hypocalcemic		Hypercalcemic	
	TransCon PTH (%)	Placebo (%)	TransCon PTH (%)	Placebo (%)	TransCon PTH (%)	Placebo (%)
Baseline	81	71	18	29	1	0
Days 2–90	88	58	4	41	8	1
Days 91–EBT	92	68	8	32	0	0

*Note*: Percentage of participants with ≥1 albumin‐adjusted serum calcium value in the normal, low, or high range by time period, *n* = 61 for TransCon PTH. *n* = 21 for placebo. Normal range for serum calcium = 8.3–10.6 mg/dL inclusive.

Abbreviations: EBT, end of blinded treatment; PTH, parathyroid hormone.

Treatment‐emergent AEs were reported in 50/61 (82%) participants treated with TransCon PTH and in 21/21 (100%) participants treated with placebo (Tables 6 and 7). Most were mild (grade 1) or moderate (grade 2) in severity. Of those in the TransCon PTH group, 30/61 (49.2%) reported a treatment‐related AE (TRAE) compared with 8/21 (38%) in the placebo group. The most common TRAEs in the TransCon PTH group were injection‐site reactions (19/61, 31.1%), hypercalcemia (6/61, 9.8%), and headache (6/61, 9.8%). No TRAEs led to treatment or trial discontinuation. Five participants (8.2%) in the TransCon PTH group reported SAEs, one of which was considered related to treatment. This participant experienced hypercalcemia in the setting of an inadvertent deviation from the titration algorithm and required hospitalization and per‐protocol interruption of treatment. The event resolved with supportive therapy, and study treatment was resumed without incident. One participant in the TransCon PTH group suffered a sudden fatal cardiac arrest deemed unrelated to study treatment. The participant was a +70‐year‐old man with postsurgical hypoparathyroidism and multiple cardiovascular risk factors, including hypertension, hyperlipidemia, and obesity. He had no known history of clinical cardiovascular disease and his course of treatment during the trial was unremarkable before the event. Anti‐TransCon PTH antibodies were detected in 8% of participants at baseline and were treatment‐emergent in 5%; anti‐PEG antibodies were detected in 17% at baseline and were treatment‐emergent in 8%; no anti‐PTH antibodies were detected. When anti‐TransCon PTH antibodies were detected, anti‐PEG antibodies were also typically detected, suggesting PEG as the epitope for anti‐TransCon PTH antibodies. None of the antibodies were neutralizing and the presence of antibodies had no evident impact on efficacy or safety outcomes.

**Table 6:** Summary: Overall Treatment‐Emergent Adverse Events

TEAE, *n* (%)^a^	TransCon PTH (*n* = 61)	Placebo (*n* = 21)
Any TEAE	50 (82)	21 (100)
Serious TEAE	5 (8)	3 (14)
Severity^b^		
Grade 1 TEAE	27 (44)	11 (52)
Grade 2 TEAE	21 (34)	9 (43)
Grade 3 TEAE	1 (2)	1 (5)
Grade 4 TEAE^c^	1 (2)	0
Treatment‐related TEAE	30 (49)	8 (38)
Serious related TEAE	1 (2)	0
TEAE related to hypercalcemia or hypocalcemia leading to ER/urgent care visit and/or hospitalization	4 (7)	2 (10)
TEAE leading to discontinuation of study drug^c^	1 (2)	2 (10)
TEAE leading to death^c^	1 (2)	0

Abbreviatiions: ER, emergency room; PTH, parathyroid hormone; TEAE, treatment‐emergent adverse event.

^a^ Percentages are calculated based on the number of participants in the safety analysis population. In the severity categories, participants are displayed for the highest severity only. TEAEs occurring before the first dose of open‐label treatment are included.

^b^ AE severity is assessed by the World Health Organization toxicity grading scale.

^c^ One participant experienced a fatal cardiac arrest (grade 4 TEAE), which subsequently led to discontinuation of the study drug and trial.

**Table 7:** Treatment‐Emergent Adverse Events in ≥5% of Participants

TEAEs by preferred term	TransCon PTH (*n* = 61)	Placebo (*n* = 21)
Participants with at least one TEAE, *n* (%)^a^	50 (82)	21 (100)
TEAEs, *n* (%)		
Injection site reaction	19 (31)	0
Headache	13 (21)	2 (10)
Hypocalcemia	6 (10)	9 (43)
Fatigue	9 (15)	5 (24)
Paresthesia	11 (18)	3 (14)
Muscle spasms	7 (12)	3 (14)
Nausea	7 (12)	2 (10)
Arthralgia	6 (10)	2 (10)
Diarrhea	6 (10)	1 (5)
Hypercalcemia	6 (10)	0
Constipation	4 (7)	1 (5)
Insomnia	4 (7)	1 (5)

Abbreviations: PTH, parathyroid hormone; TEAE, treatment‐emergent adverse event.

^a^ Percentages are calculated based on the number of participants in the safety analysis population.

## Discussion

In this randomized trial of women and men with chronic hypoparathyroidism, PTH replacement therapy with TransCon PTH robustly improved and maintained mean serum calcium levels in the normal range at all study visits, while allowing independence from conventional therapy. Treatment with TransCon PTH additionally resulted in significant improvement in physical functioning and well‐being, as well as potential longer‐term benefits evident in the normalization of urinary calcium excretion. TransCon PTH treatment was safe and well‐tolerated, with no participants discontinuing therapy because of a related adverse event. Unique to this trial was the ability to titrate the dose of TransCon PTH offering an individualized approach to improve treatment outcomes.

Significantly more participants treated with TransCon PTH (79%, *n* = 48/61) than placebo (5%, *n* = 1/21) met the composite primary endpoint (*p* < 0.0001). The most common reason participants treated with TransCon PTH did not meet the composite primary endpoint was an isolated albumin‐adjusted serum calcium lab value below the normal range at week 26, though most often still within the range targeted in clinical practice (i.e., in the low normal range or slightly below normal [≤0.5 mg/dL below normal]).^(^ ^3^ ^)^ The majority of TransCon PTH‐treated participants (93%, *n* = 57/61) in this trial achieved independence from both active vitamin D and therapeutic doses of elemental calcium, which was associated with a significant reduction in daily pill burden. This significant reduction in conventional therapy was accompanied by a lower incidence of laboratory‐measured hypocalcemia in those receiving TransCon PTH. This is in contrast to a greater incidence of hypocalcemia and an overall decline in serum calcium with placebo treatment, possibly attributable in part to protocol‐driven down titration of conventional therapy. In addition to maintaining normocalcemia, serum phosphate and calcium × phosphate product remained within the normal range in the TransCon PTH group. Consistent with initial findings from the phase 2 PaTH Forward trial,^(^ ^13^ ^)^ mean 1,25‐dihydroxyvitamin D levels also remained within the normal range with TransCon PTH independent from conventional therapy. These results suggest that TransCon PTH restored and sustained endogenous production of calcitriol and underscore the importance of PTH replacement therapy for treating the underlying hormone deficiency of hypoparathyroidism.

In the phase 2 PaTH Forward trial, TransCon PTH was associated with normalization of renal absorption of filtered calcium from baseline (415 mg/24 h) to week 26 (178 mg/24 h).^(^ ^13^ ^)^ These two large clinical trials, which enrolled a population of individuals with chronic hypoparathyroidism, have shown and replicated a significant reduction in urine calcium excretion with PTH replacement therapy. It may be that continuous exposure of the renal tubule to circulating active PTH levels throughout 24 hours, as occurs with the sustained release of active PTH from the TransCon PTH prodrug, is necessary to achieve decreased urinary calcium excretion. Renal complications of hypoparathyroidism and its conventional therapy are associated with long‐term morbidity in this population.^(^ ^11^ ^)^ The ability to reduce the filtered load of calcium by reducing conventional therapy and also by promoting tubular calcium reabsorption may lead to reductions in the risk of nephrolithiasis, nephrocalcinosis, and ultimately chronic kidney disease prevalent in individuals with hypoparathyroidism.^(^ ^1^, ^11^, ^15^ ^)^ Additionally, the normalization of serum phosphate and calcium × phosphate product may reduce the long‐term risk of extra‐renal calcifications.^(^ ^15^, ^23^ ^)^

Individuals with hypoparathyroidism often experience physical and cognitive symptoms that impact multiple dimensions of health and well‐being that persist despite conventional therapy and improvements in serum calcium.^(^ ^1^, ^7^, ^24^ ^)^ Previous open‐label and observational studies have shown that individuals with hypoparathyroidism treated with PTH report improvements in QoL.^(^ ^25^, ^26^ ^)^ To the authors' knowledge, the 4‐week, double‐blind, placebo‐controlled, phase 2 PaTH Forward trial was the first trial in individuals with chronic hypoparathyroidism to show that a PTH replacement therapy significantly enhanced HRQoL.^(^ ^13^ ^)^ In the 26‐week PaTHway trial, TransCon PTH therapy significantly improved disease‐specific measures of symptoms, functioning, and well‐being across all HPES‐Symptom (physical and cognitive) and HPES‐Impact (physical functioning and daily life) domains. These results demonstrate that TransCon PTH therapy has the potential to provide significant and sustained improvements not only in clinical outcomes but also in HRQoL and symptoms in adults with hypoparathyroidism, which were observed as early as 10 weeks after initiation of treatment in the PaTHway trial.

TransCon PTH was well‐tolerated with a safety profile as expected. Most AEs were mild or moderate in severity and no TRAEs led to withdrawal from the trial. There was a numerically greater incidence of hypercalcemia with TransCon PTH within the first 3 months of treatment and a consistently greater incidence of hypocalcemia with placebo throughout the trial. The majority of participants (57 of 61) receiving TransCon PTH remained on a stable dose of study drug for a minimum of 4 weeks before assessment of the primary endpoint, supporting a durable treatment effect.

Potential limitations of this trial merit consideration. Real‐time measurement of serum calcium was not available, and therefore, symptoms suggestive of calcium abnormalities could not be universally confirmed with laboratory studies before treatment with PRN conventional therapy. Participants and investigators were blinded to the allocation of study drug but were aware of calcium and active vitamin D doses for safety reasons. The substantial reduction in conventional therapy in participants treated with TransCon PTH has the potential for a degree of functional unblinding and may have contributed to changes in patient‐reported outcomes. Evaluation of long‐term effects of treatment and event‐driven evaluation of end‐organ outcomes were limited by trial duration, though data from the ongoing open‐label extensions of the PaTH Forward and PaTHway trials will permit further assessment.

The participant population of the PaTHway trial is consistent with previous clinical trials in hypoparathyroidism and representative of previously reported cohorts of this disease, supporting the generalizability of these results to the broader population of adults with chronic hypoparathyroidism.^(^ ^27^, ^28^, ^29^ ^)^ PaTHway had a suitable number of participants for the study of this rare disease, was adequately powered for assessment of the primary efficacy endpoint, and had high participant retention. Validated outcome measures were used to evaluate the impact of TransCon PTH on a wide range of clinical aspects of hypoparathyroidism, as well as HRQoL and disease‐specific functioning and well‐being. Compliance with study drug and co‐administered conventional therapy were confirmed by patient‐recorded daily diaries. No participant discontinued the study drug because of a TRAE or drug intolerance.

The PaTHway trial met all primary and key secondary endpoints with statistically significant differences from placebo. In this randomized trial of adults with hypoparathyroidism dependent on conventional therapy, the ability to maintain normocalcemia while gaining independence from active vitamin D and elemental calcium (>600 mg/d) was significantly greater among the participants who received TransCon PTH compared with those receiving placebo. TransCon PTH treatment was well‐tolerated, normalized mean 24‐hour urine calcium excretion, and led to improvements in HRQoL and hypoparathyroidism‐related symptoms, functioning, and well‐being. The results of the PaTHway trial both confirm and extend findings from initial reports of the efficacy of treatment with TransCon PTH in restoring physiological functions of PTH in individuals with chronic hypoparathyroidism.

## Disclosures

AAK: Research investigator for Alexion, Amgen, Ascendis, Chugai, Radius, Takeda, and Ultragenyx. MRR: Research investigator for Ascendis Pharma Inc. PS: Research investigator for Ascendis Pharma A/S. TJV: Advisory board member for Takeda; receives consulting fees from Ascendis Pharma Inc.; and research investigator for Ascendis Pharma Inc. DMS: Advisory board member for Amolyt; receives consulting fees from Takeda; and research investigator for Ascendis Pharma Inc. CG: Advisory board member for Novo Nordisk; grant recipient from Novo Nordisk; and research investigator for Shire and Ascendis Pharma Inc. AP: Research investigator for Ascendis Pharma A/S. CM: Research investigator for Ascendis Pharma A/S. BLC: Advisory board member for Takeda, Shire, PARADIGHM Registry Steering Committee, Ascendis Pharma Inc; grant recipient from Takeda, Shire, Chugai, and Ascendis Pharma Inc.; and research investigator for Ascendis Pharma Inc. LGA: Research investigator for Ascendis Pharma Inc and Takeda; and speaker for Abbvie and Clarus. LCH: Advisory board member for Amgen, Kiowa Kirin International, and UCB. LK: Advisory board member for Alexion; research investigator for Ascendis Pharma Inc; and speaker for Radius, Amgen, and Alexion. SP, XA, WE, ARS, JU, CTS, and ADS: Employee of Ascendis Pharma Inc. LR: Advisory board member for Takeda and Amolyt; grant recipient from Kyowa Kirin and Takeda; and research investigator for Ascendis Pharma A/S, Takeda, and Kyowa Kirin.

## Author Contributions

**Aliya Aziz Khan:** Investigation; writing – original draft; writing – review and editing. **Mishaela R. Rubin:** Investigation; writing – review and editing. **Peter Schwarz:** Investigation; writing – review and editing. **Tamara J. Vokes:** Investigation; writing – review and editing. **Dolores Shoback:** Investigation; writing – review and editing. **Claudia Gagnon:** Investigation; writing – review and editing. **Andrea Palermo:** Investigation; writing – review and editing. **Claudio Marcocci:** Investigation; writing – review and editing. **Bart L. Clarke:** Investigation; writing – review and editing. **Lisa G. Abbott:** Investigation; writing – review and editing. **Lorenz Hofbauer:** Investigation; writing – review and editing. **Lynn Kohlmeier:** Investigation; writing – review and editing. **Susanne Pihl:** Conceptualization; methodology; writing – original draft; writing – review and editing. **Xuebei An:** Conceptualization; data curation; formal analysis; methodology; writing – original draft; writing – review and editing. **Walter Frank Eng:** Methodology; project administration; writing – original draft; writing – review and editing. **Alden R. Smith:** Conceptualization; methodology; writing – original draft; writing – review and editing. **Jenny Ukena:** Conceptualization; methodology; project administration; writing – original draft; writing – review and editing. **Christopher Tate Sibley:** Conceptualization; data curation; methodology; writing – original draft; writing – review and editing. **Aimee D. Shu:** Conceptualization; data curation; methodology; writing – original draft; writing – review and editing. **Lars Rejnmark:** Investigation; writing – review and editing.

## Supporting information


**Fig. S1.** Titration algorithm.

## Data Availability

The data sets generated during and/or analyzed during the present study are not publicly available but are available from the corresponding author on reasonable request.
