# Supplementary material for: Efficacy and Safety of Parathyroid Hormone Replacement With TransCon PTH in Hypoparathyroidism: 26‐Week Results From the Phase 3 PaTHway Trial
Source: J Bone Miner Res. 2022 Nov 12;38(1):14–25. doi: 10.1002/jbmr.4726 (PMC10099823; doi:10.1002/jbmr.4726)
Supplement: Supplementary file 1 — Fig. S1. Titration algorithm. [file JBMR-38-14-s001.docx]

**Supplemental Figure 1. Titration Algorithm**


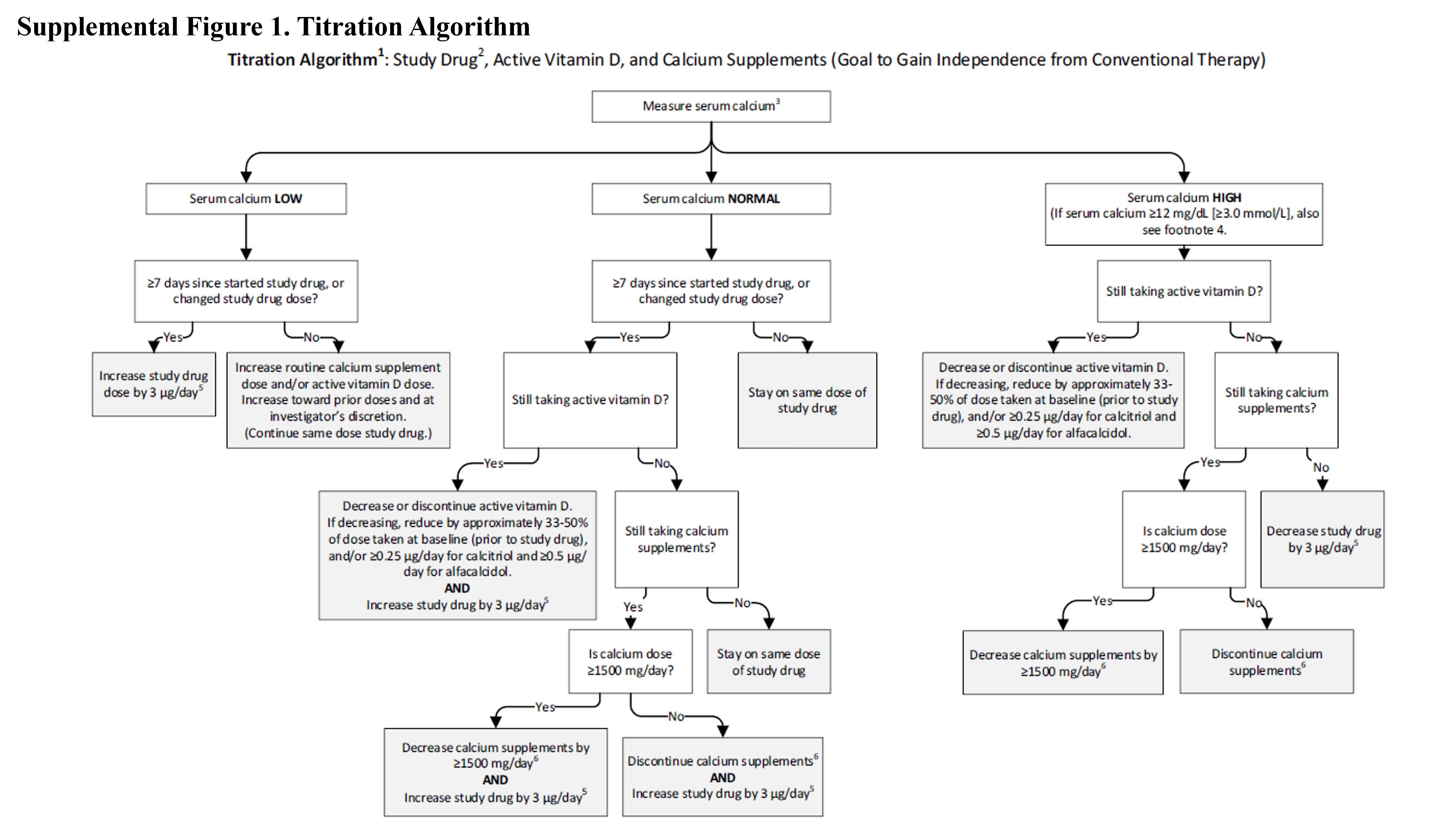


^1^ At Visit 1 (Week 0, Day 1), start study drug at 18 μg/day and decrease active vitamin D dose by 33-50% (e.g., skip second dose of the day if taking BID, and skip final dose of the day if taking TID).

^2^ *Study drug* refers to TransCon PTH or placebo.

^3^ *sCa* refers to either albumin-adjusted sCa and/or ionized calcium. For the purposes of this trial, the normal ranges are: albumin-adjusted sCa 8.3-10.6 mg/dL (2.07-2.64 mmol/L); ionized calcium 1.16-1.32 mmol/L.

^4^ If albumin-adjusted sCa ≥12.0 mg/dL (3.00 mmol/L) or ionized calcium ≥1.50 mmol/L, hold study drug for approximately 2-3 days. Remember to resume study drug therapy afterwards. Also reduce study drug, active vitamin D, or calcium as per algorithm.

^5^ Through Week 52: check sCa within 7-14 days after any changes in study drug dose; standing calcium, standing vitamin D doses; or sCa outside the normal range. A scheduled visit or LV within 7-14 days meets this requirement. When scheduled study visits occur less frequently (e.g. 13 weeks apart) then an ULV should be pursued. After Week 52 of the trial check sCa within 7-31 days after any changes in study drug dose.

^6^ The goal is to demonstrate independence from therapeutic doses of calcium supplements. In case needed to meet recommended dietary intake of calcium, it is permitted to take calcium supplements ≤600 mg/day as a nutritional supplement for the sake of reaching the recommended dietary intake.
